# Supraphysiological Estrogen Induces Endometrial Impairments via Mitochondrial ROS-Driven NLRP3 Inflammasome Activation

**DOI:** 10.3390/antiox15091170

**Published:** 2026-09-15

**Authors:** Yiran Sun, Hao Yang, Lu Lu, Jiangxue Cai, Chenxi Liu, Jianguo Zhu, Bin He

**Affiliations:** 1 Key Laboratory of Animal Physiology & Biochemistry, Ministry of Agriculture and Rural Affairs, College of Veterinary Medicine, Nanjing Agricultural University, Nanjing 210095, China; 2023107005@stu.njau.edu.cn (Y.S.); yanghao93@stu.njau.edu.cn (H.Y.); 2023007047@stu.njau.edu.cn (L.L.); caijx@hebau.edu.cn (J.C.); 2023107006@stu.njau.edu.cn (C.L.); zhujianguo@stu.njau.edu.cn (J.Z.); 2 MOE Joint International Research Laboratory of Animal Health and Food Safety, Nanjing Agricultural University, Nanjing 210095, China

**Keywords:** estrus synchronization, estradiol, NLRP3 inflammasome, mitochondrial ROS, gilt

## Abstract

Exogenous gonadotropin-based estrus synchronization is widely used in livestock reproduction. However, this hormonal treatment has been associated with adverse reproductive outcomes, suggesting that it may alter the uterine environment and endometrial receptivity, although the underlying molecular mechanism remains poorly understood. Here, we investigated the endometrial responses to gonadotropin-induced estrus synchronization using both *in vivo* and *in vitro* models. We found that gonadotropin treatment caused structural and inflammatory changes in the endometrium of both pigs and mice, accompanied by elevated circulating estradiol (E_2_) levels and enhanced NLRP3 inflammasome-related signaling. In mice, gonadotropin treatment also altered the expression of MUC1, Hand2, and HoxA11, indicating disruption of molecular features associated with endometrial receptivity. In porcine endometrial epithelial cells, supraphysiological E_2_ exposure induced mitochondrial oxidative stress, characterized by increased mitochondrial reactive oxygen species production, loss of mitochondrial membrane potential, increased cellular mtDNA abundance, and oxidative DNA damage. E_2_ treatment further promoted NLRP3 inflammasome-associated signaling and ASC speck formation, whereas antioxidant N-acetyl-L-cysteine treatment attenuated ASC speck formation. Collectively, these findings support a link between high E_2_ exposure, mitochondrial oxidative stress, and enhanced NLRP3 inflammasome signaling in endometrial epithelial cells. Our results suggest that an E_2_–mitochondrial oxidative stress–NLRP3 axis may contribute to gonadotropin-associated endometrial dysfunction and provide a basis for further investigation of antioxidant and inflammasome-targeted strategies in livestock reproduction.

## 1. Introduction

In animal reproductive management, exogenous gonadotropins are widely used in estrus synchronization and timed artificial insemination protocols to stimulate follicular growth and maturation [1]. Controlled ovarian stimulation is a central component of assisted reproductive technology (ART), in which exogenous gonadotropins are administered to induce multifollicular development before oocyte retrieval and embryo transfer [2]. Although these interventions substantially improve the availability of mature oocytes, ovarian stimulation also generates a non-physiological endocrine environment and has been associated with altered endometrial development and adverse reproductive outcomes in both experimental animals and humans [3,4]. In women undergoing controlled ovarian stimulation, endometrial gene-expression profiles during the receptive phase differ substantially from those observed during natural cycles, indicating that ovarian stimulation can modify the molecular program underlying endometrial receptivity [5,6]. Compared with naturally conceived pregnancies, pregnancies achieved through ART have been associated with an elevated risk of antepartum hemorrhage and miscarriage [7,8]. Similar adverse reproductive phenotypes have also been observed in gonadotropin-stimulated mouse [9,10] and pig models [1,11], suggesting that the uterine consequences of ovarian stimulation represent an important component of the reproductive response to exogenous gonadotropin administration.

Successful implantation requires precise temporal coordination between a developmentally competent blastocyst and a receptive endometrium. The acquisition of endometrial receptivity is governed by the coordinated and sequential actions of ovarian steroid hormones, particularly estradiol (E_2_) and progesterone (P_4_) [12], which regulate epithelial proliferation, differentiation, stromal signaling, and implantation-associated remodeling [13]. Ovarian stimulation can perturb this physiological endocrine balance by producing supraphysiological concentrations of ovarian steroids [14], although the magnitude and relative contribution of E_2_ and P_4_ vary according to the stimulation protocol and physiological context [15,16]. Human endometrial transcriptomic studies have demonstrated marked differences between natural and stimulated cycles, and supraphysiological E_2_ exposure has been associated with altered expression of genes involved in endometrial receptivity and a potential shift in the window of implantation [5,6]. Accordingly, excessive estrogenic signaling represents a plausible contributor to the uterine effects of ovarian stimulation, but the cellular pathways linking supraphysiological E_2_ exposure to endometrial dysfunction remain incompletely understood.

Sterile inflammation is a hallmark of various estrogen-associated endometrial disorders, including endometrial cancer, endometrial hyperplasia [17], and endometriosis [18]. Unlike classical inflammation, sterile inflammation is not triggered by pathogens but is primarily initiated by damage-associated molecular patterns (DAMPs) released from damaged tissues or necrotic cells [19]. The NLRP3 inflammasome is a multiprotein signaling complex comprising the sensor NLRP3, the adaptor protein ASC, and the effector pro-Caspase-1 [20]. Upon activation, NLRP3 recruits ASC, which undergoes oligomerization and forms supramolecular ASC specks that facilitate pro-Caspase-1 recruitment and activation, ultimately promoting the maturation of IL-1β and IL-18 and, under appropriate conditions, gasdermin D-dependent pyroptotic signaling [21], serving as a core mediator of sterile inflammatory reactions [22]. Our previous results show that angiogenin ameliorates porcine endometritis via NLRP3 inflammasome inhibition [23]. Importantly, NLRP3 activation should not be regarded as intrinsically pathological in the endometrium. Cheng et al. demonstrated that NLRP3 expression is physiologically elevated in the human endometrium during the mid-secretory phase and is transcriptionally induced by E_2_ through estrogen receptor β (ERβ); NLRP3 also promoted endometrial epithelial remodeling and embryo adhesion in their experimental models [24]. These findings suggest that NLRP3 signaling may exert dose-, timing-, and context-dependent functions in the endometrium. Whereas appropriately regulated NLRP3 activity may participate in implantation-associated remodeling, excessive or sustained inflammasome activation in the presence of cellular stress may instead contribute to a maladaptive sterile inflammatory response.

Accumulating evidence indicates that E_2_ influences the onset and progression of female reproductive diseases by regulating the NLRP3 inflammasome [25]. Mitochondrial dysfunction provides a potential mechanistic link between excessive estrogenic stimulation and aberrant inflammasome activation. Estrogen receptors, including ERα and ERβ, have been identified within mitochondria and can modulate mitochondrial gene transcription and respiratory function [26]. In addition, estrogen metabolism can generate redox-active catechol estrogen metabolites whose oxidation–reduction cycling contributes to ROS production [27,28]. Thus, although physiological estrogen signaling can support cellular and mitochondrial homeostasis, excessive estrogenic exposure may disturb redox balance and promote oxidative stress in susceptible cellular contexts [29]. Mitochondria are particularly important in this regard because disruption of mitochondrial function can increase mitochondrial ROS (mtROS) generation and alter mitochondrial membrane potential [30]. Damaged, ROS-generating mitochondria have been established as potent upstream signals for NLRP3 inflammasome activation [19,31], and mitochondrial damage can additionally generate mitochondrial DNA (mtDNA)-associated danger signals capable of promoting inflammasome activation [32]. Whether such mitochondrial stress links supraphysiological E_2_ exposure to excessive NLRP3 inflammasome assembly in the endometrial epithelium, however, remains poorly defined.

In the present study, we investigated whether gonadotropin-induced estrus synchronization is associated with endometrial inflammatory responses and whether high E_2_ exposure is sufficient to promote mitochondrial oxidative stress and NLRP3 inflammasome activation in porcine endometrial epithelial cells. Using porcine and murine *in vivo* models together with an *in vitro* Porcine endometrial epithelial cells (PEECs) model, we evaluated endometrial morphology, inflammatory signaling, receptivity-associated molecular features, mitochondrial redox status, and NLRP3 inflammasome assembly. We hypothesized that excessive E_2_ exposure perturbs mitochondrial redox homeostasis, resulting in increased mtROS and mitochondrial stress that promote aberrant NLRP3 inflammasome assembly. By distinguishing physiological NLRP3 signaling from excessive activation associated with mitochondrial stress, this study aimed to provide a mechanistic framework for understanding the inflammatory component of gonadotropin-associated endometrial dysfunction.

## 2. Materials and Methods

### 2.1. Animal Models

All Landrace × Yorkshire × Duroc gilts were housed in an enclosed facility. Twelve cycling gilts were selected and randomly assigned to two groups: a control group consisting of naturally cycling gilts, and a PMSG treatment group. Gilts in the PMSG group were orally administered altrenogest (2 mg/day) for 18 days. After a 42-h withdrawal period, the gilts received an intramuscular injection of 1000 IU PMSG. All gilts were humanely euthanized 3 days later, and uterine tissues were collected for subsequent molecular analyses.

Wild-type C57BL/6J mice were purchased from GemPharmatech (Nanjing, China). Female mice aged 6–8 weeks and weighing 18–22 g were randomly assigned to two groups: control group and PMSG group. Mice in the PMSG group received an intraperitoneal injection of 20 IU pregnant mare serum gonadotropin (PMSG, Ningbo Second Hormone Factory, Ningbo, China), whereas mice in the control group received 1.25 IU PMSG to synchronize the estrous cycle. After 46–48 h, all mice were intraperitoneally injected with 5 IU human chorionic gonadotropin (hCG, Ningbo Second Hormone Factory, Ningbo, China). Mice were housed under specific pathogen-free conditions at the Laboratory Animal Center of Nanjing Agricultural University, with ad libitum access to food and water, under a 12-h light/12-h dark cycle at 22–25 °C and 40–60% relative humidity. All mice were euthanized 3.5 days after PMSG administration, corresponding to the expected implantation period, and uterine tissues were collected for subsequent experiments.

### 2.2. Histology and Immunohistochemical Analysis

For histological evaluation, fixed uterine tissues were dehydrated, embedded in paraffin and sectioned at a thickness of 5 μm. Sections were stained with hematoxylin/eosin (H&E), and histomorphological changes were examined under a microscope (BX63F OLYMPUS Micro Image System, OLYMPUS, Tokyo, Japan). Neutrophils were identified according to their characteristic multilobed nuclei and cellular morphology. For each animal, 6 randomly selected non-overlapping fields were analyzed. The number of neutrophils within the endometrial region was counted using QuPath-0.5.0, and neutrophil density was expressed as cells/mm^2^. Quantification was performed by an investigator blinded to the treatment groups.

For immunohistochemical staining, 5-μm-thick uterine sections were deparaffinized in xylene and rehydrated using a graded ethanol series. Antigen retrieval was performed in 10 mM citrate buffer (pH 6.0) at 95 °C for 15 min, after which the sections were allowed to cool to room temperature and washed twice with distilled water. Endogenous peroxidase activity was blocked by incubation with 3% H_2_O_2_ for 20 min at room temperature in the dark, followed by blocking with 5% bovine serum albumin (BSA) at 37 °C for 1 h. The sections were then incubated overnight at 4 °C with rabbit anti-NLRP3 antibody (1:800, #15101; Cell Signaling Technology, Danvers, MA, USA) or rabbit anti-MUC1 antibody (1:500, A19081; ABclonal, Wuhan, China). After incubation with the appropriate HRP-conjugated goat anti-rabbit IgG secondary antibody (RGAU011; Proteintech, Chicago, IL, USA), immunoreactivity was visualized using diaminobenzidine (DAB), followed by hematoxylin counterstaining. Three animals were randomly selected from each group for histological and histomorphometric analyses.

### 2.3. Cell Culture

The PEECs (iCell-0048a) and specific culture medium (iCell-0048a-001b) were purchased from iCell Bioscience Inc. (Shanghai, China). Cells were maintained at 37 °C in a humidified atmosphere containing 5% CO_2_. PEECs were treated with E_2_ (E2758, Sigma, Burlington, MA, USA) at concentrations of 10^−8^, 10^−7^, or 10^−6^ M for 24 h. Cells cultured without E_2_ served as the control group. To attenuate oxidative stress, PEECs were pretreated with 10 mM N-acetyl-L-cysteine (NAC, A9165, Sigma, Burlington, MA, USA) for 30 min before E_2_ treatment.

### 2.4. Western Blotting

Uterine tissue samples (30 mg) were homogenized in RIPA buffer (P0013B, Beyotime, Shanghai, China) supplemented with 1× protease inhibitor (K1007, APExBIO, Houston, TX, USA) and 1× phosphatase inhibitor (K1015, APExBIO, Houston, TX, USA). Cell samples were lysed by sonication. The lysates were incubated on ice for 30 min and then centrifuged at 12,000× *g* for 10 min at 4 °C. The resulting supernatants were collected, and protein concentrations were determined using a bicinchoninic acid (BCA) assay kit (GK10009, GLPBIO, Montclair, CA, USA). Equal amounts of protein were separated by sodium dodecyl sulfate–polyacrylamide gel electrophoresis (SDS-PAGE) and transferred onto polyvinylidene fluoride (PVDF) membranes (IPVH00010, Millipore, Burlington, MA, USA) at 100 V for 1.5 h. The membranes were blocked with 5% non-fat milk for 2 h at room temperature and then incubated overnight at 4 °C with gentle agitation with the following primary antibodies: rabbit anti-NLRP3 antibody (1:1000, ab283819, abcam, Cambridge, UK), rabbit anti-GSDMD antibody (1:1000, ab219800; Abcam, Cambridge, UK), mouse anti-Caspase-1 antibody (1:1000, AG-20B-0042; AdipoGen, Epalinges, Switzerland), rabbit anti-ASC (#67824, Cell Signaling Technology, Danvers, MA, USA), and rabbit anti-α-Tubulin (1:1000, AC007, ABClonal, Wuhan, China). Protein bands were visualized using the VersaDoc 4000MP system (Bio-Rad, Hercules, CA, USA), and the band intensities were quantified using ImageJ software 1.53c.

### 2.5. RNA Extraction and RT-qPCR

Total RNA was extracted from uterine tissues and PEECs using TRIzol reagent (R401, Vazyme, Nanjing, China) according to the manufacturer’s instructions. Total RNA was reverse-transcribed into cDNA using a TransScript^®^ Uni All-in-One First-Strand cDNA Synthesis SuperMix for qPCR kit (AU341-02, TransGen Biotech, Beijing, China). Each qPCR reaction was performed in a final volume of 10 μL, containing 5 μL of 2× PerfectStart^®^ Green qPCR SuperMix, 0.2 μL of forward primer (10 μM), 0.2 μL of reverse primer (10 μM), 1 μL of cDNA, 0.2 μL of 50× Universal Passive Reference Dye, and 3.4 μL of nuclease-free water. All reactions were performed in triplicate. Gene expression levels were normalized to β-actin, and relative expression was calculated using the 2^−ΔΔCt^ method. Primer sequences for mouse and pig genes are listed in Table 1 and Table 2, respectively.

### 2.6. Immunofluorescence

PEECs were fixed with 4% paraformaldehyde for 10 min and permeabilized with 0.3% Triton X-100 for 1 h. After blocking with 5% BSA for 10 min, the cells were incubated overnight with anti-ASC antibody (A1170, ABClonal, Wuhan, China). The cells were then incubated with Alexa Fluor 488-conjugated goat anti-rabbit IgG secondary antibody (A-11008, Invitrogen, Carlsbad, CA, USA). Nuclei were counterstained with DAPI (C1005, Beyotime, Shanghai, China) for 5 min.

### 2.7. Biochemical Assays

The superoxide indicator MitoSOX is cell-permeant and specifically targets mitochondria in live cells. Once inside the mitochondria, the MitoSOX probe is oxidized specifically by superoxide anions, and the oxidized product subsequently binds to nucleic acids, exhibiting strong red fluorescence. We incubated the cells with PBS containing 4 μM MitoSOX (M36008, Thermo Fisher, Waltham, MA, USA), then harvested them with FACS buffer (2% FBS and 2 mM EDTA in PBS) and analyzed them with flow cytometry (BD Biosciences, San Jose, CA, USA) after 20 min at 37 °C.

To determine serum E_2_ levels, blood samples were centrifuged at 3000 rpm for 10 min at 4 °C. The resulting serum was collected and stored at −20 °C until analysis. Serum E_2_ concentrations were determined by radioimmunoassay at Beijing North Biotechnology Institute Co., Ltd. (Beijing, China).

For extracting total DNA, cell samples were lysed in TES buffer (1 M Tris-HCl pH 8.0, 0.5 M EDTA pH 8.0, 5 M NaCl, 10% SDS with triply distilled water) containing proteinase K (BL104A, Biosharp, Beijing, China). Cell lysates were then collected and incubated at 55 °C overnight. DNA extraction phenol was added, and the mixture was thoroughly mixed by inversion. After centrifugation, DNA extraction phenol containing isoamyl alcohol and chloroform (phenol:chloroform:isoamyl alcohol = 25:24:1) was added to the supernatant and mixed thoroughly by inversion. Following a second centrifugation, 1/10 volume of 3 M sodium acetate (NaAc) and an equal volume of isopropanol were added to the supernatant. After centrifugation, the resulting pellet was washed with 75% ethanol. Finally, the pellet was dissolved in TE buffer (1 M Tris-HCl pH 8.0, 0.5 M EDTA pH 8.0, prepared with triple-distilled water) containing RNase A (BS109, Biosharp, Beijing, China) to obtain high-quality genomic DNA. Mitochondrial DNA was quantified by qPCR using primers targeting cytochrome c oxidase subunit 1 (Cox1) or a mitochondrial DNA region lacking nuclear mitochondrial DNA sequence (NUMT) insertions. Nuclear DNA genes 18S ribosomal RNA and β2-microglobulin (B2m) were used as reference genes for normalization. Primer sequences are listed in Table 2.

### 2.8. Mitochondrial Membrane Potential (Δψm) Analysis

JC-1 is a cationic fluorescent dye widely used for detecting ΔΨm. In healthy mitochondria, JC-1 accumulates in the mitochondrial matrix and forms aggregates that emit intense red fluorescence. Conversely, in damaged mitochondria with decreased membrane potential, JC-1 remains in monomeric form and yields green fluorescence. PEECs were incubated with complete culture medium containing 1× JC-1 dye (KTA4001, Abbkine, Wuhan, China) for 20 min at 37 °C. Images were then acquired within 1 h using an inverted fluorescence microscope (DMI6000B, Leica, Wetzlar, Germany). Mitochondrial membrane potential was quantified as the ratio of red-to-green fluorescence intensity using ImageJ.

### 2.9. Enzyme-Linked Immunosorbent Assay (ELISA)

Cell samples were collected, and 8-hydroxy-2′-deoxyguanosine (8-OHdG) levels were measured using a commercial assay kit (E-EL-0028, Elabscience, Wuhan, China). Standard solutions and diluted test samples were added to the antibody-precoated microplate, followed by the addition of biotinylated antibody working solution and incubation at 37 °C for 45 min. After washing, HRP-conjugated streptavidin was added and incubated at 37 °C for 30 min. Following another wash step, substrate solution was added and incubated in the dark before the reaction was terminated. The optical density (OD) of each well was measured immediately at a wavelength of 450 nm using a microplate reader (Synergy 2, BioTek, Winooski, VT, USA). All experiments were performed with three independent biological replicates, and each replicate included three technical replicates.

### 2.10. Statistical Analysis

All data are presented as the mean ± standard error of the mean (SEM) and were analyzed using GraphPad Prism 9. Before analysis, the normal distribution of data was validated using the Shapiro–Wilk test. Comparisons between the two groups were performed using an unpaired two-tailed Student’s t-test for normally distributed data. Comparisons among three or more groups were performed using one-way analysis of variance (ANOVA), followed by Tukey’s multiple comparison test. The *p*-value results are denoted by asterisks in the figures (* *p* < 0.05, ** *p* < 0.01, *** *p* < 0.001, and **** *p* < 0.0001).

## 3. Results

### 3.1. Estrus Synchronization Induces Endometrial Injury and Alters Receptivity-Associated Molecular Markers

**Figure 1:**
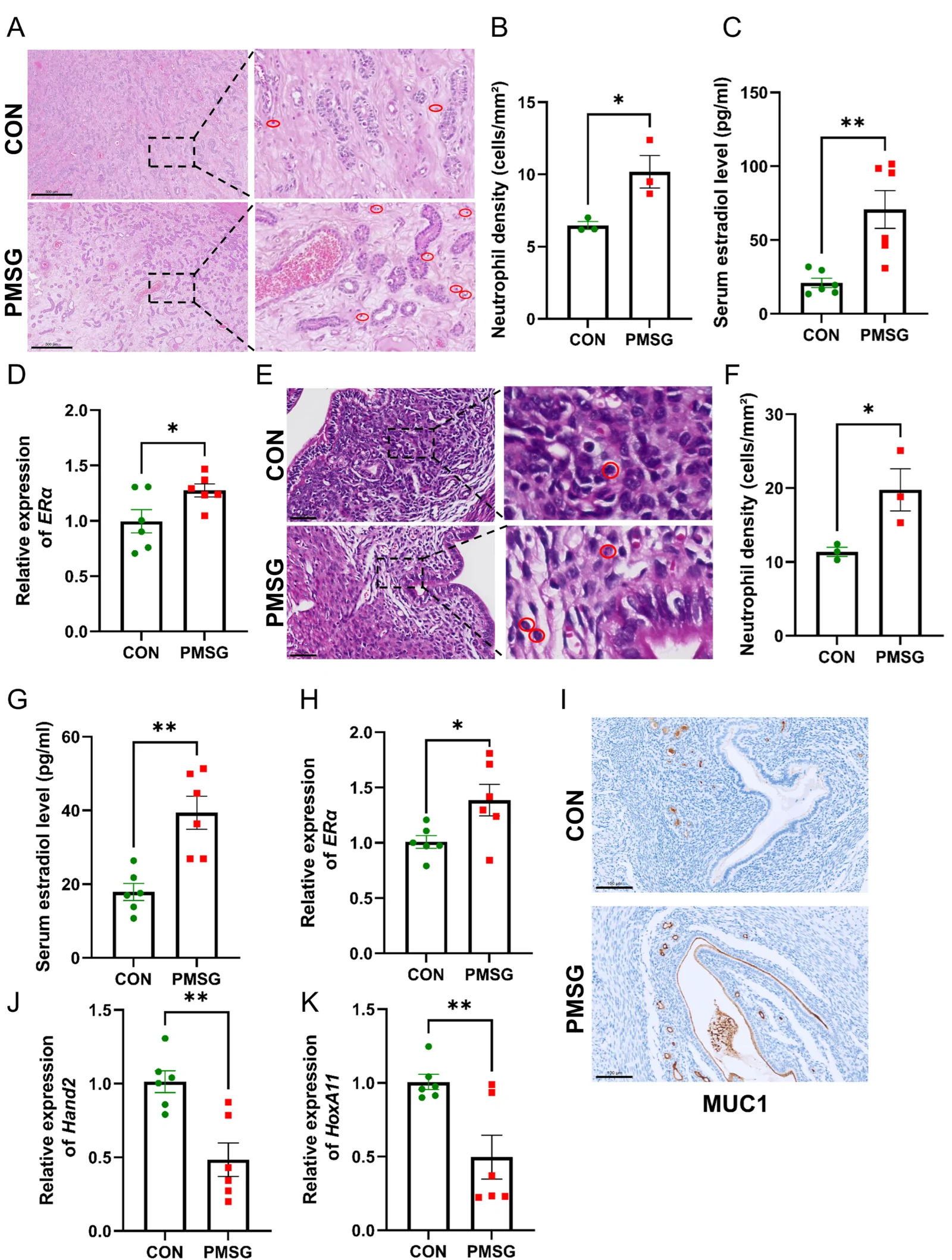
Estrus synchronization causes structural endometrial damage and altered receptivity-associated markers. (**A**) Representative H&E-stained sections of uterine tissue from gilts. Scale bar = 500 μm. Red circles indicate areas of neutrophil infiltration (*n* = 3). (**B**) Neutrophil density (cells/mm^2^) in the gilt endometrium (*n* = 3). (**C**) Serum estradiol (E_2_) concentration in gilts (*n* = 6). (**D**) Relative mRNA expression level of *ERα* in porcine uterine tissue (*n* = 6). (**E**) Representative H&E-stained sections of mouse uterine tissue. Scale bar = 50 μm. Red circles indicate areas of neutrophil infiltration. (**F**) Neutrophil density (cells/mm^2^) in the mouse endometrium (*n* = 3). (**G**) Serum E_2_ concentration in mice (*n* = 6). (**H**) Relative mRNA expression level of *ERα* in mouse uterine tissue (*n* = 6). (**I**) Immunohistochemical staining of MUC1 in the mouse uterus. Scale bar = 100 μm. (**J**,**K**) Relative mRNA expression levels of *Hand2* and *HoxA11* in mouse uterine tissue (*n* = 6). Data are presented as mean ± SEM. * *p* < 0.05, ** *p* < 0.01.

To determine whether gonadotropin-induced estrus synchronization alters the endometrial microenvironment in gilts and mice, we first evaluated uterine histopathology, circulating E_2_ levels, and estrogen signaling in both species. Histological examination of uterine tissues from PMSG-treated gilts revealed marked pathological features, including diffuse stromal edema, extensive neutrophil infiltration within the endometrial glands and lumen, and focal detachment of the luminal epithelium (Figure 1A). Quantitative analysis showed a marked increase in endometrial neutrophil density in the PMSG group (*p* < 0.05, Figure 1B). Serum E_2_ concentrations were significantly elevated in PMSG-treated gilts (*p* < 0.01, Figure 1C), accompanied by a significant upregulation of *ERα* mRNA expression in uterine tissues (*p* < 0.05, Figure 1D). Similarly, PMSG-treated mice displayed pronounced uterine edema and substantial neutrophil infiltration (Figure 1E), with a significant increase in endometrial neutrophil density (*p* < 0.05; Figure 1F). Serum E_2_ concentrations were also significantly elevated in PMSG-treated mice (*p* < 0.01; Figure 1G), and *ERα* mRNA levels were markedly upregulated in the uterus (*p* < 0.05; Figure 1H). In summary, these findings indicate that PMSG treatment was associated with elevated circulating E_2_ levels and endometrial histopathological alterations in both gilts and mice.

The expression of key endometrial receptivity-associated markers was also altered following estrus synchronization in mice. Immunohistochemical analysis showed increased MUC1 expression in the endometrial epithelium (Figure 1I). In addition, RT-qPCR analysis demonstrated that the mRNA expression levels of *Hand2* and *HoxA11* were significantly downregulated following estrus synchronization (*p* < 0.01; Figure 1J,K). These findings indicate that gonadotropin-induced estrus synchronization is associated with altered expression of molecular markers related to endometrial receptivity.

### 3.2. Estrus Synchronization Activates the NLRP3 Inflammasome in the Endometrium

**Figure 2:**
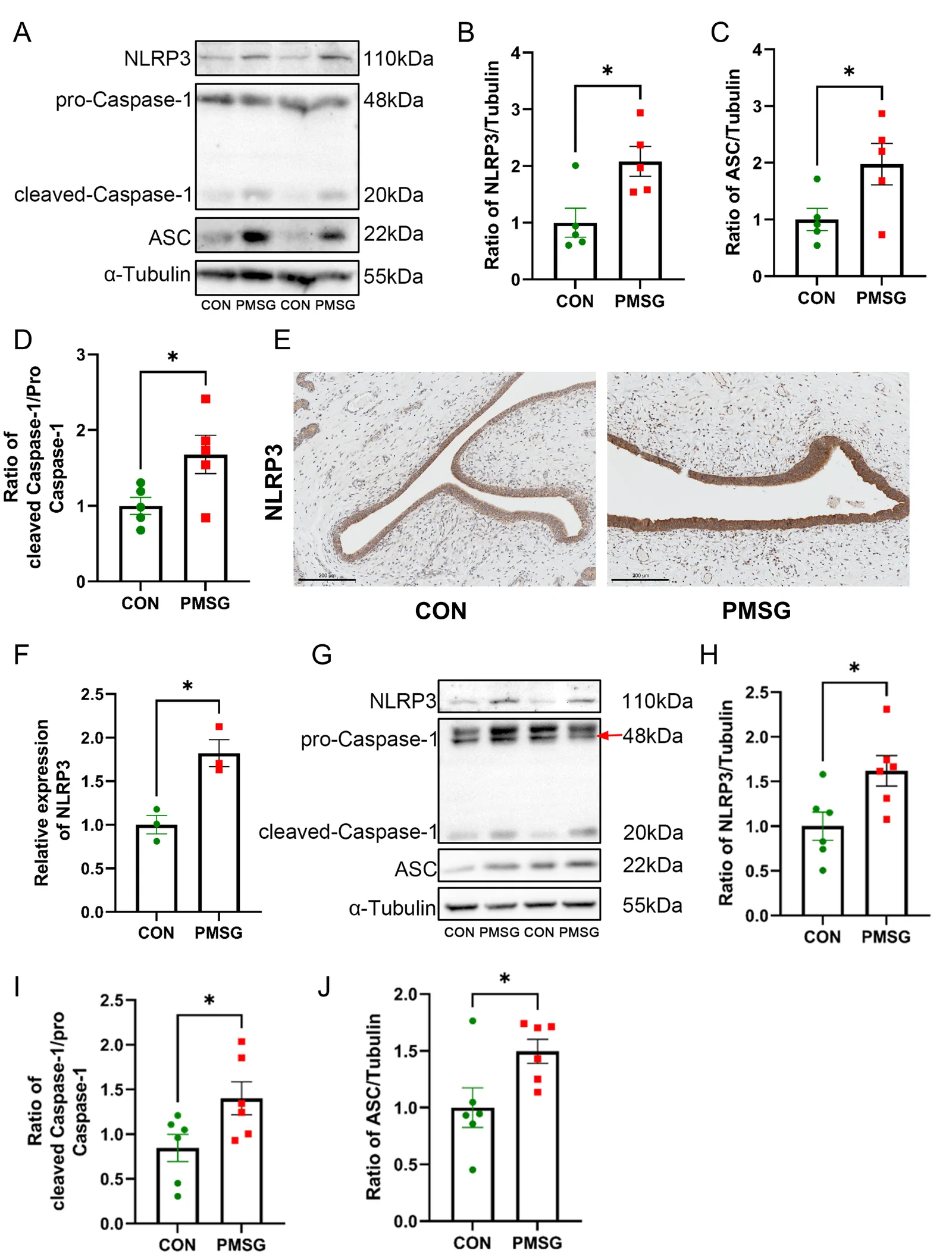
Estrus synchronization activates the NLRP3 inflammasome in the endometrium. (**A**) Representative immunoblots of NLRP3, pro-Caspase-1, cleaved Caspase-1, ASC, and α-tubulin in gilt uterine tissue. (**B**–**D**) Densitometric analyses of NLRP3/α-tubulin, ASC/α-tubulin, and cleaved Caspase-1/pro-Caspase-1, respectively (*n* = 5). (**E**,**F**) Representative NLRP3 immunohistochemical images (**E**) and average optical density analysis (**F**) in gilt uterine tissue. Scale bars = 200 μm (*n* = 3). (**G**) Representative immunoblots of NLRP3, pro-Caspase-1, cleaved Caspase-1, ASC, and α-tubulin in mouse uterine tissue. The red arrow identifies the pro-Caspase-1 band. (**H**–**J**) Densitometric analyses of NLRP3/α-tubulin, cleaved Caspase-1/pro-Caspase-1, and ASC/α-tubulin, respectively (*n* = 6). Data are presented as mean ± SEM. * *p* < 0.05.

To determine whether NLRP3 inflammasome activation was associated with endometrial injury induced by estrus synchronization, we examined the expression of NLRP3 inflammasome-related proteins. Western blot analysis revealed significantly elevated protein levels of NLRP3, ASC, and cleaved Caspase-1 in uterine tissues from PMSG-treated gilts compared with controls (*p* < 0.05; Figure 2A–D). Immunohistochemical analysis further showed increased NLRP3 protein expression in the endometrial epithelium of PMSG-treated gilts (*p* < 0.05; Figure 2E,F). Similar changes were observed in mice. PMSG treatment was associated with significantly increased protein levels of NLRP3, cleaved Caspase-1, and ASC in uterine tissues (*p* < 0.05, Figure 2G–J). Collectively, these findings indicate that gonadotropin-induced estrus synchronization is associated with activation of the NLRP3 inflammasome in the endometrium.

### 3.3. Estrogen Activates the NLRP3 Inflammasome in Endometrial Epithelial Cells

**Figure 3:**
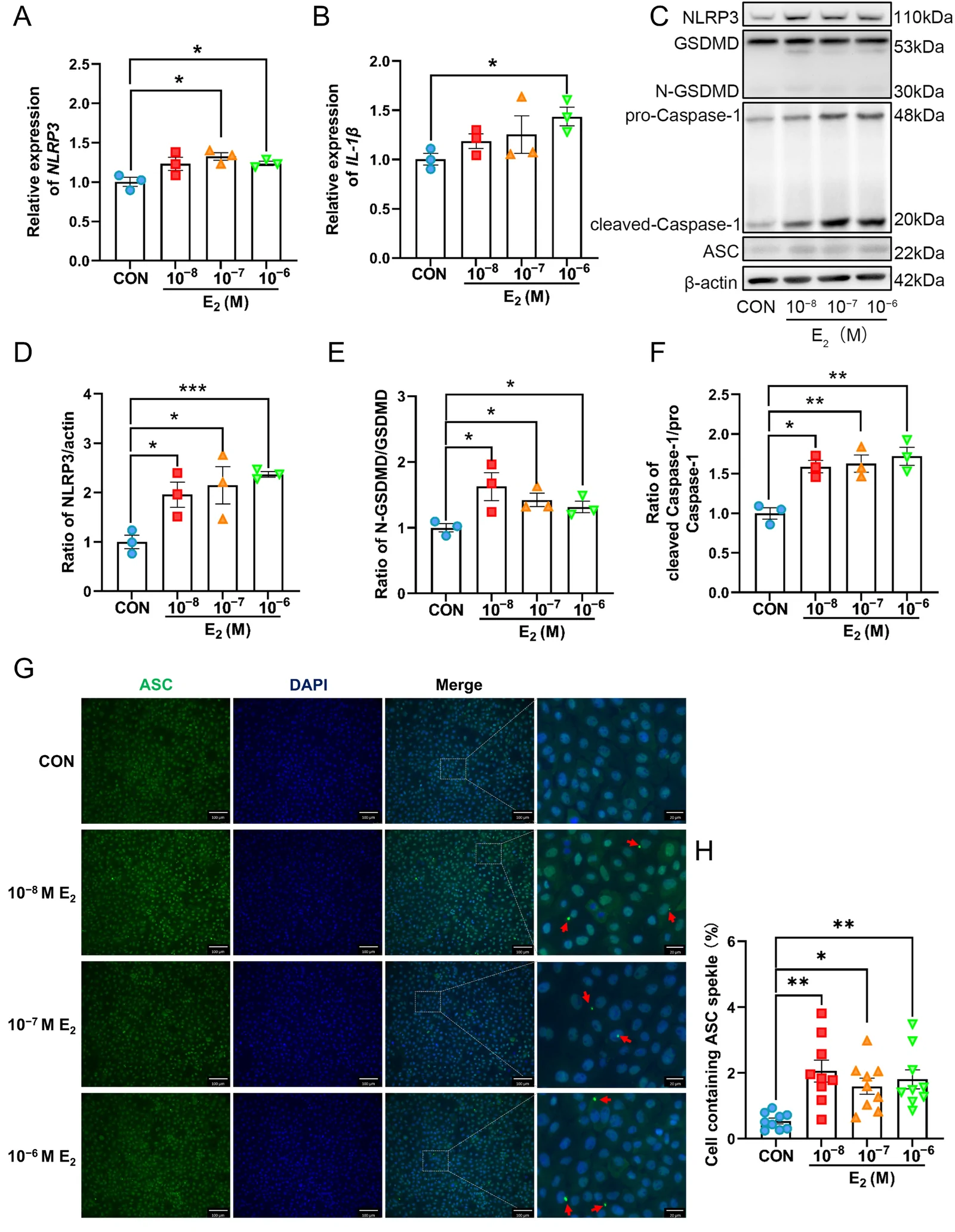
E_2_-induced NLRP3 inflammasome activation in porcine endometrial epithelial cells. Porcine endometrial epithelial cells (PEECs) were treated with 10^−8^, 10^−7^, or 10^−6^ M E_2_ for 24 h. (**A**,**B**) Relative mRNA expression levels of *NLRP3* and *IL-1β* in PEECs. (**C**) Representative immunoblots of NLRP3, GSDMD, N-GSDMD, pro-Caspase-1, cleaved Caspase-1, ASC, and β-actin. (**D**–**F**) Densitometric analyses of NLRP3/β-actin, N-GSDMD/GSDMD, and cleaved Caspase-1/pro-Caspase-1, respectively. (**G**,**H**) Representative immunofluorescence images (**G**) and quantification (**H**) of ASC speck formation in PEECs stimulated with E_2_ (10^−8^, 10^−7^, or 10^−6^ M) for 24 h. Scale bar = 100 μm (Left), Scale bar = 20 μm (Right). Red arrows indicate ASC specks. Data are presented as mean ± SEM (*n* = 3). * *p* < 0.05, ** *p* < 0.01, *** *p* < 0.001.

To investigate whether elevated E_2_ contributes to NLRP3 inflammasome activation in endometrial epithelial cells, PEECs were treated with increasing concentrations of E_2_ (10^−8^, 10^−7^, and 10^−6^ M). RT-qPCR analysis showed that treatment with 10^−7^ M and 10^−6^ M E_2_ for 24 h significantly increased *NLRP3* mRNA expression (*p* < 0.05; Figure 3A), whereas *IL-1β* mRNA expression was significantly elevated only at 10^−6^ M E_2_ (*p* < 0.05; Figure 3B). Western blot analysis revealed that E_2_ treatment upregulated the protein levels of NLRP3, cleaved Caspase-1, and cleaved GSDMD in a concentration-dependent manner, whereas ASC protein expression remained unchanged compared with the control group (*p* < 0.05; Figure 3C–F). ASC is an adaptor protein that links activated NLRP3 to pro-Caspase-1, and its activation is characterized primarily by oligomerization and speck formation rather than by an increase in total protein abundance. Therefore, to further assess inflammasome assembly, ASC speck formation was examined by immunofluorescence. The proportion of cells containing ASC specks increased progressively with increasing E_2_ concentrations (*p* < 0.05; Figure 3G,H). These findings indicate that elevated E_2_ promotes NLRP3 inflammasome activation in PEECs in vitro.

### 3.4. E_2_-Induced NLRP3 Inflammasome Activation Is Mediated by Mitochondrial Dysfunction

**Figure 4:**
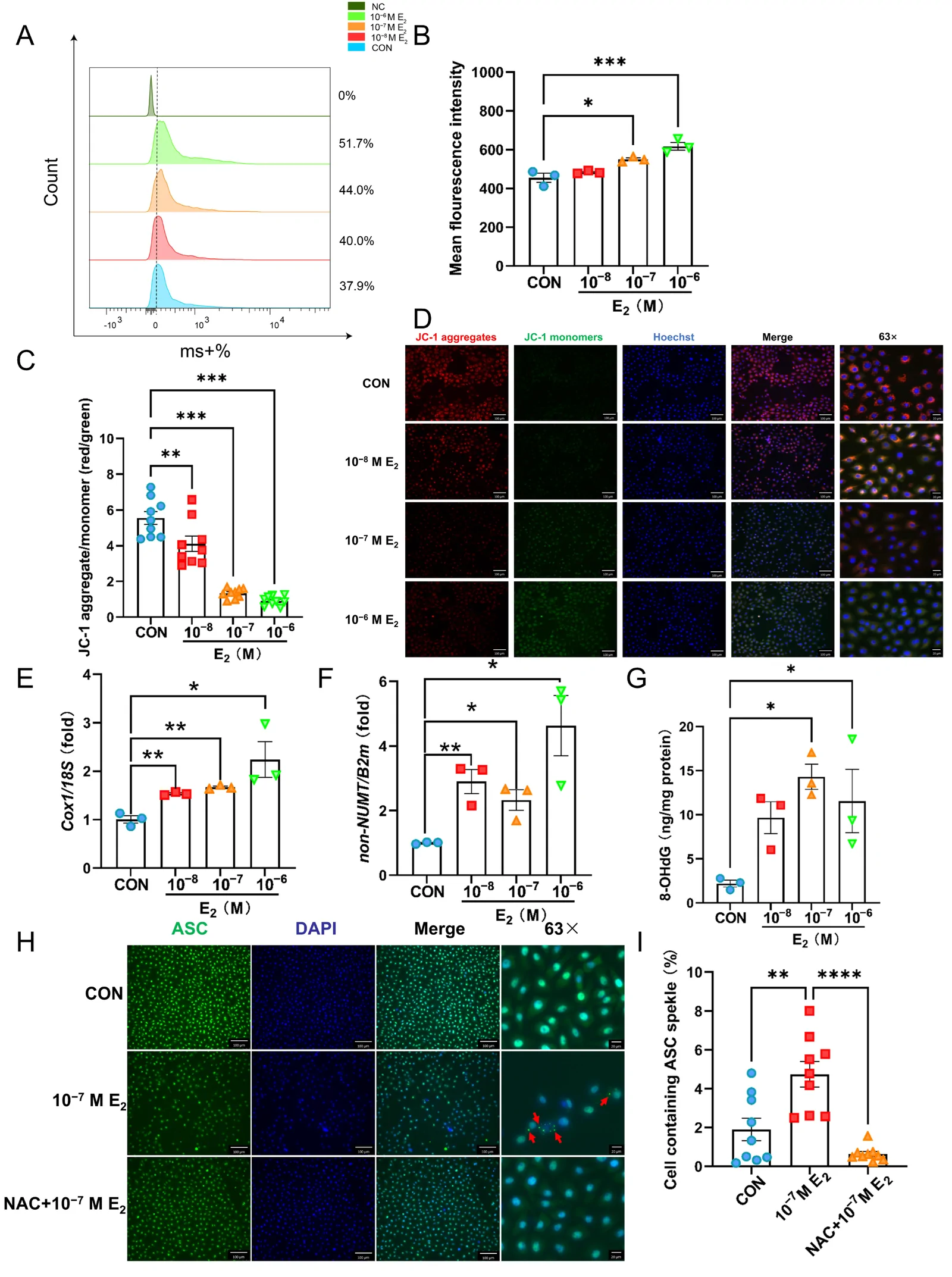
E_2_-induced NLRP3 inflammasome activation via mitochondrial dysfunction. PEECs were treated with 10^−8^, 10^−7^, or 10^−6^ M E_2_ for 24 h. (**A**) Representative flow cytometry histograms of MitoSOX fluorescence, with the percentages of MitoSOX-positive cells indicated. NC denotes the unstained negative control. (**B**) Mean MitoSOX fluorescence intensity. (**C**,**D**) Quantification of the red-to-green fluorescence intensity ratio (**C**) and representative fluorescence images (**D**) of JC-1-stained PEECs for assessment of mitochondrial membrane potential. Scale bar = 100 μm (Left), Scale bar = 20 μm (Right). (**E**,**F**) qPCR analysis of the Cox1 mtDNA/18S nuclear DNA ratio (**E**) and non-NUMT mtDNA/B2m nuclear DNA ratio (**F**). (**G**) 8-OHdG levels in PEECs measured by ELISA following E_2_ treatment (10^−8^, 10^−7^, or 10^−6^ M) for 24 h. (**H**,**I**) Representative immunofluorescence images (**H**) and quantification (**I**) of ASC speck formation in PEECs pretreated with NAC for 0.5 h before E_2_ treatment for 24 h. Scale bar = 100 μm (Left), Scale bar = 20 μm (Right). Red arrows indicate the representative ASC specks. Data are presented as mean ± SEM (*n* = 3). * *p* < 0.05, ** *p* < 0.01, *** *p* < 0.001, **** *p* < 0.0001.

To further elucidate the mechanism by which E_2_ induces NLRP3 inflammasome activation, mitochondrial reactive oxygen species (mtROS) levels were assessed by flow cytometry. The mean fluorescence intensity of mtROS was significantly increased in PEECs treated with 10^−7^ M and 10^−6^ M E_2_ compared with the control group, whereas no significant difference was observed following treatment with 10^−8^ M E_2_ (*p* < 0.05; Figure 4A,B). Given that mitochondrial damage is a key upstream event in NLRP3 inflammasome activation, the ΔΨm was subsequently assessed using JC-1 staining. E_2_ treatment resulted in a concentration-dependent decrease in the red-to-green fluorescence intensity ratio, consistent with a progressive loss of Δψm (*p* < 0.01; Figure 4C,D). We next examined changes in mtDNA abundance and oxidative damage following E_2_ treatment. qPCR analysis showed significant increases in the Cox1/18S and non-NUMT/B2m ratios following E_2_ treatment (*p* < 0.05; Figure 4E,F), indicating increased cellular mtDNA abundance. Furthermore, ELISA showed a significant increase in 8-hydroxy-2′-deoxyguanosine (8-OHdG), a well-established biomarker of oxidative DNA damage, following E_2_ treatment (*p* < 0.05; Figure 4G), supporting enhanced oxidative DNA damage. To further investigate whether ROS are responsible for NLRP3 inflammasome activation, cells were pretreated with the antioxidant NAC for 0.5 h before E_2_ treatment. Immunofluorescence analysis showed that E_2_ treatment increased ASC speck formation, an effect that was markedly attenuated by NAC pretreatment (*p* < 0.01; Figure 4H,I). Taken together, these findings indicate that E_2_ induces mitochondrial oxidative stress and loss of mitochondrial membrane potential, and that ROS contribute to E_2_-induced NLRP3 inflammasome activation in PEECs.

## 4. Discussion

In this study, gonadotropin-based estrus synchronization induced marked structural damage and inflammatory responses in the endometrium of both pigs and mice, together with increased circulating E_2_ levels and activation of NLRP3 inflammasome signaling. In mice, PMSG treatment altered the expression of MUC1, Hand2, and HoxA11, suggesting disruption of molecular features associated with endometrial receptivity. In PEECs, supraphysiological E_2_ increased mtROS production, reduced mitochondrial membrane potential, and enhanced NLRP3, cleaved Caspase-1, cleaved GSDMD, and ASC speck formation. Moreover, NAC markedly attenuated E_2_-induced ASC speck formation. Together, these findings support a potential link between gonadotropin-associated endocrine changes, mitochondrial oxidative stress, and enhanced NLRP3 inflammasome signaling in the endometrium.

Previous studies have shown that ovarian stimulation can alter the developmental program of the endometrium. Transcriptomic analyses of the human endometrium have revealed substantial differences in the expression of receptivity-related genes between natural and stimulated cycles [5,6]. Consistent with these findings, our mouse model showed increased MUC1 expression together with reduced *Hand2* and *HoxA11* expression, suggesting disruption of molecular processes required for the establishment of endometrial receptivity. MUC1 is a highly glycosylated epithelial surface protein with anti-adhesive properties, and its downregulation during the implantation period in mice facilitates interaction between the blastocyst and the luminal epithelium [33]. Hand2 is a progesterone-responsive stromal transcription factor that restrains estrogen-driven epithelial proliferation by suppressing fibroblast growth factor signaling. Accordingly, uterine-specific deletion of Hand2 results in persistent epithelial proliferation and impaired implantation [34]. HoxA11 also contributes to endometrial differentiation and implantation-related stromal function and is regulated by ovarian steroid hormones [35]. Taken together, these changes indicate that PMSG-treated mice exhibit a molecular profile consistent with reduced endometrial receptivity. Successful embryo implantation depends on the precise and coordinated actions of ovarian hormones. As key upstream regulators, estrogen and progesterone activate multiple downstream signaling pathways that drive the structural and functional remodeling required for the establishment of a receptive endometrium [36]. However, serum progesterone and other ovarian endocrine factors were not comprehensively assessed in the present study, and estrogen signaling was not selectively inhibited in vivo. Therefore, the uterine effects of PMSG cannot be attributed solely to E_2_. Further studies are therefore needed to define the causal hierarchy of this signaling pathway.

The role of NLRP3 in the endometrium also appears to be highly environment-dependent. Although excessive inflammasome activation is widely associated with sterile inflammatory injury, NLRP3 signaling is not necessarily detrimental to endometrial function. Cheng et al. reported that NLRP3 is physiologically expressed in the human mid-secretory endometrium, where its transcription can be upregulated by E_2_ through estrogen receptor β (ERβ), thereby promoting epithelial remodeling and embryo adhesion [24]. These findings suggest that appropriately regulated NLRP3 activity may contribute to the normal establishment of endometrial receptivity. Our results should therefore be interpreted in the context of dose, timing, and physiological state. In our in vivo models, enhanced NLRP3 signaling was accompanied by neutrophil infiltration and structural damage, whereas supraphysiological E_2_ exposure in PEECs induced mitochondrial oxidative stress, caspase-1 activation, GSDMD cleavage, and increased ASC speck formation. Thus, our findings do not imply that NLRP3 activation is inherently pathological. Rather, they support the view that excessive or stress-associated inflammasome activation may represent a maladaptive form of this pathway, whereas tightly regulated NLRP3 signaling can also participate in normal reproductive physiology.

Notably, total ASC protein abundance differed among the experimental models, increasing in porcine and mouse uterine tissues but remaining unchanged in PEECs. This apparent discrepancy is not necessarily inconsistent with inflammasome activation, because total ASC abundance and ASC functional assembly represent distinct biological processes. ASC acts as an adaptor protein that links activated inflammasome sensors to pro-Caspase-1 and, upon inflammasome activation, undergoes oligomerization and spatial redistribution into supramolecular ASC specks that provide a platform for pro-Caspase-1 recruitment and activation [37]. Consistent with this mechanism, E_2_ markedly increased ASC speck formation in PEECs despite no significant change in total ASC protein abundance. The differential changes in total ASC abundance among porcine and murine uterine tissues and PEECs may reflect species-specific responses, temporal regulation of inflammasome components, and differences in cellular composition. Whole uterine tissue contains multiple cell populations, including epithelial, stromal, vascular, and immune cells, whereas PEECs represent a relatively homogeneous epithelial cell model. Therefore, the increase in total ASC observed in uterine tissue may partly reflect contributions from non-epithelial cell populations, including infiltrating immune cells. However, because the cellular source of ASC was not specifically determined in the present study, this interpretation remains to be established experimentally.

Mitochondrial stress provides a plausible mechanistic link between high E_2_ exposure and enhanced inflammasome signaling. The effects of estrogen on mitochondrial function are complex and appear to be concentration-dependent. Both ERα and ERβ are localized in mitochondria, where they can regulate mitochondrial transcription and respiratory activity [26]. In addition, E_2_ can be hydroxylated to catechol estrogens, including 2-hydroxyestradiol and 4-hydroxyestradiol, which can undergo sequential one-electron oxidation to semiquinone radicals and quinones. Redox cycling between the catechol and quinone species can generate ROS, including superoxide and hydrogen peroxide, thereby contributing to oxidative damage [38,39]. In the present study, supraphysiological E_2_ exposure caused a concentration-dependent increase in mitochondrial ROS and a reduction in mitochondrial membrane potential in PEECs, indicating disruption of mitochondrial redox homeostasis under these conditions. Damaged, ROS-producing mitochondria are well-established upstream signals for NLRP3 inflammasome activation. Previous studies have also shown that dietary supplementation with NAC can suppress excessive ERK phosphorylation and alleviate abnormal endometrial epithelial proliferation in gilts [1]. Consistent with an involvement of oxidative stress in inflammasome activation, NAC pretreatment markedly reduced E_2_-induced ASC speck formation in our cell model. However, NAC is a broad-spectrum antioxidant rather than a mitochondria-specific ROS scavenger [40]. Therefore, these findings support a role for ROS in promoting inflammasome assembly but do not establish mitochondrial ROS as the sole upstream signal.

In summary, our findings show that gonadotropin-induced endocrine changes are associated with endometrial inflammation and enhanced NLRP3 inflammasome signaling, while supraphysiological E_2_ is sufficient to induce mitochondrial oxidative stress and promote inflammasome assembly in porcine endometrial epithelial cells. These results do not suggest that NLRP3 activation is inherently detrimental; rather, they indicate that excessive inflammasome activation under conditions of mitochondrial oxidative stress may disrupt endometrial homeostasis. This study therefore provides a basis for further investigation of mitochondrial redox regulation and NLRP3 signaling as potential targets for improving reproductive outcomes following gonadotropin stimulation.

## 5. Conclusions

Overall, exogenous gonadotropin-mediated estrus synchronization was associated with elevated circulating E_2_ and endometrial inflammatory injury in pigs and mice, together with altered receptivity-associated molecular features in mice. In vitro, high-dose E_2_ induced mitochondrial oxidative stress and enhanced NLRP3 inflammasome signaling in porcine endometrial epithelial cells, supporting a potential estrogen–mitochondria–NLRP3 axis in gonadotropin-associated endometrial dysfunction. These findings provide a basis for further evaluating mitochondrial oxidative stress and NLRP3 inflammasome signaling as potential targets to improve reproductive outcomes following estrus synchronization in livestock.

## Figures and Tables

**Table 1:** Nucleotide sequences of mouse primers.

Target Genes	Accession No.	Primer Sequences (5′ to 3′)	
*ERα*	XM_011243068.4	F: CCTCCCGCCTTCTACAGGT	R: CACACGGCACAGTAGCGAG
*Hand2*	NM_010402.4	F: GGAAGGCGAGATGAGTCTGG	R: CATCTCCGGGTGGCCAATAA
*HoxA11*	NM_010450.4	F: TTTGATGAGCGTGGTCCCTG	R: AGGAGTAGGAGTATGTCATTGGG
β-actin	NM_007393.5	F: TTCTTTGCAGCTCCTTCGTT	R: ATGGAGGGGAATACAGCCC

**Table 2:** Nucleotide sequences of pig primers.

Target Genes	Accession No.	Primer Sequences (5′ to 3′)	
*ERα*	XM_021083018.1	F: TCAACTGGGCAAAGAGGGTG	R: CTCCACCATTCCCTCGACAC
*NLRP3*	XM_021078925.1	F: ATTACCCGCCCGAGAAAGG	R: TCGCAGCAAAGATCCACACAG
*IL-1β*	NM_001302388.2	F: GTGGCAGGACCTACACTCTTC	R: TTCCTTCAGAATGCCGTCCT
*COX1*	KF888634.1	F: TGGTGCCTGAGCAGGAATAGTG	R: ATCATCGCCAAGTAGGGTTCCG
*18S*	NR_046261.1	F: CTGAGAAACGGCTACCACATC	R: AATTACAGGGCCTCGAAAGAG
*non-NUMT*	NC_000845.1	F: CAGACGAGCTACCCATGAGC	R: ACAACCAGCTATCACCAGGC
*B2m*	NM_213978.1	F: GATCAGTATAGCTGCCGCGT	R: ATGCTCAGATTCGGTTGGCA
β-actin	XM_021086047.1	F: CTCCATCATGAAGTGCGACGT	R: GTGATCTCCTTCTGCATCCTGTC

## Data Availability

The data presented in this study are available within the article.

## Abbreviations

The following abbreviations are used in this manuscript:

NLRP3	NOD-, LRR- and pyrin domain-containing protein 3
ASC	Apoptosis-associated speck-like protein containing CARD
Caspase-1	Cysteine-aspartic acid protease 1
E_2_	17β-Estradiol
mtDNA	Mitochondrial DNA
ART	Assisted reproductive technology
DAMP	Damage-associated molecular pattern
IL-1β	Interleukin-1β
IL-18	Interleukin-18
ERβ	Estrogen receptor β
ERα	Estrogen receptor α
PMSG	Pregnant mare serum gonadotropin
hCG	Human chorionic gonadotropin
PEEC	Porcine endometrial epithelial cells
NAC	N-acetyl-L-cysteine
MUC1	Mucin 1
GSDMD	Gasdermin D
ROS	Reactive oxygen species
Cox1	Mitochondrial cytochrome c oxidase subunit 1
Non-NUMT	Non-nuclear mitochondrial DNA segments
18S	18S ribosomal RNA
B2m	beta-2-microglobulin
Hand2	Heart and Neural Crest Derivatives-expressed 2
HoxA11	Homeobox-A11
P_4_	Progesterone
mtROS	Mitochondrial reactive oxygen species
ΔΨm	Mitochondrial membrane potential
8-OHdG	8-Hydroxy-2′-deoxyguanosine
